# Supplementary material for: Identification of Potential p38γ Inhibitors via In Silico Screening, In Vitro Bioassay and Molecular Dynamics Simulation Studies
Source: Int J Mol Sci. 2023 Apr 17;24(8):7360. doi: 10.3390/ijms24087360 (PMC10139033; doi:10.3390/ijms24087360)
Supplement: Supplementary file 1 [file ijms-24-07360-s001.zip › ijms-2330566-supplementary.pdf]

**Table of Contents:**

**Figure S1.** The workflow for the p38γ inhibitor identification process. The numbers in brackets correspond to the total number of compounds at each step.

**Figure S2.** PCA biplot mapping the chemical space of compounds in the training set (red circles) and test set (blue circles).

**Figure S3.** (A) Plots of predicted and experimental values for the LibSVM with CDK fingerprint model model (Red: training set, blue: test set, green: 10-fold CV). (B) ROC curves of the LibSVM with CDK fingerprint model.

**Figure S4.** Comparison of the experimental ANP conformation (red) and its re-docking pose predicted by (A) SMINA docking by smina (green), dkoes (blue), vinardo (magenta), and ad4 (cyan) scoring functions; and (B) GOLD docking by CHEMPLP (green), GoldScore (blue), ChemScore (magenta), and ASP (cyan) scoring functions.

**Figure S5.** The root mean square deviation (RMSD) values between experimental pose of ANP and its docking pose predicted by SMINA with the scoring functions (blue) and GOLD with the scoring functions (green).

**Figure S6.** The ROC curves of p38γ dataset run by (A) SMINA docking with smina (blue), dkoes (orange), vinardo (green), and ad4 (red) scoring functions; (B) GOLD docking with CHEMPLP (cyan), GoldScore (violet), ChemScore (olive), and ASP (crimson) scoring functions; and (C) GOLD docking with ASP+GoldScore (turquoise), ASP+GoldScore+CHEMPLP (brown), ASP+CHEMPLP (magenta) consensus scoring.

**Figure S7.** Receiver operating characteristic (ROC) curves of the query verification for (A) ROCS matching; (B) EON comparison.

**Figure S8.** 2D chemical structure of (A) compound 1; (B)compound 2; (C) compound 3; (D) pirfenidone; and (E) PIK75.

**Figure S9.** Root mean square deviation (RMSD) plots of (A) protein backbone; (B) binding sites of protein during 100 ns MD simulations. Red stands for compound 2 complex; blue stands for apo protein.

**Figure S10.** (A) Eigenvalues of the first 60 eigenvectors derived from PCA of each simulation trajectories of p38γ protein. (B) Projection of the motion of combined trajectories along the PC1 and PC2. Red stands for compound 2 complex; blue stands

for apo protein.

**Table S1.** Performance summary of QSAR models for predicting $pIC_{50}$.

**Table S2.** Checklist of hit compounds and their predicted information.

**Table S3.** 16 hit compounds list.

**Table S4.** The values of RMSD and Rg, and number of hydrogen bonds of systems during simulation.

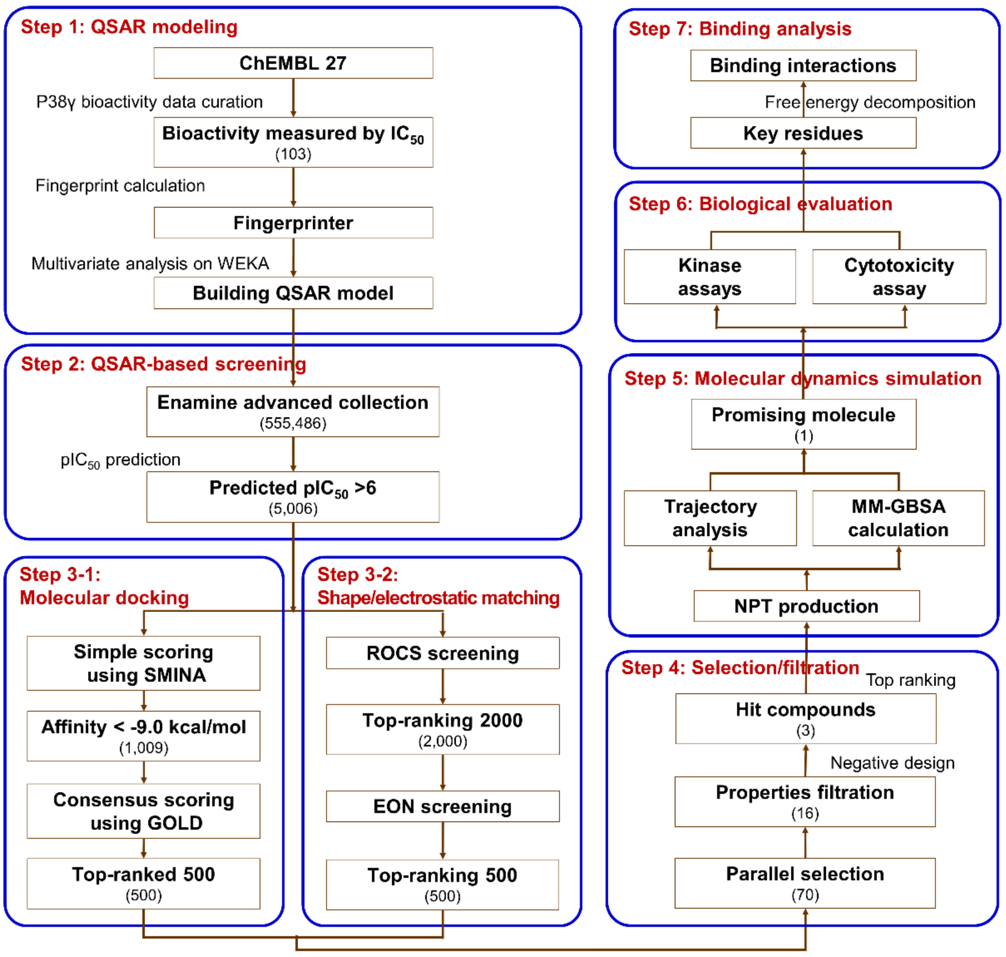


**Figure S1.** The workflow for the p38γ inhibitor identification process. The numbers in brackets correspond to the total number of compounds at each step.

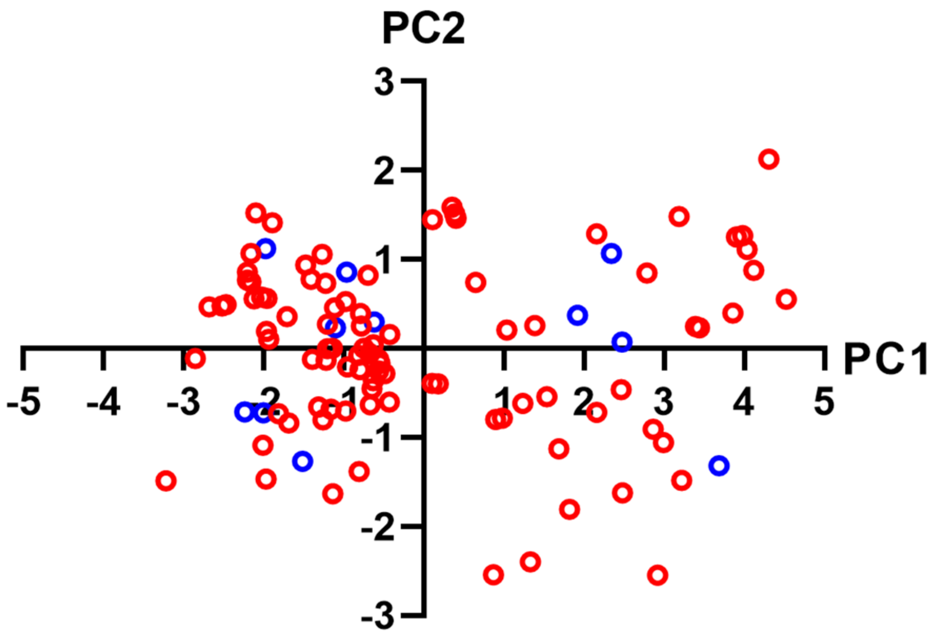


**Figure S2.** PCA biplot mapping the chemical space of compounds in the training set (red circles) and test set (blue circles).

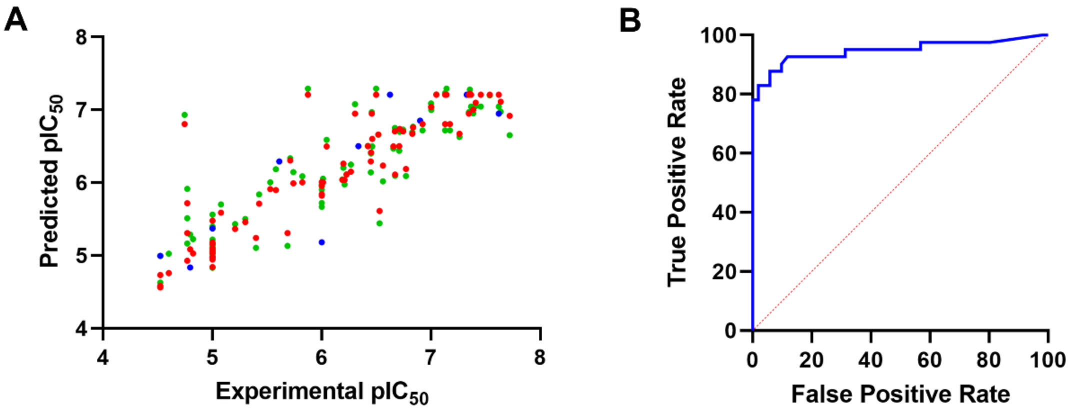


**Figure S3.** (**A**) Plots of predicted and experimental values for the LibSVM with CDK fingerprint model model (Red: training set, blue: test set, green: 10-fold CV). (**B**) ROC curves of the LibSVM with CDK fingerprint model.

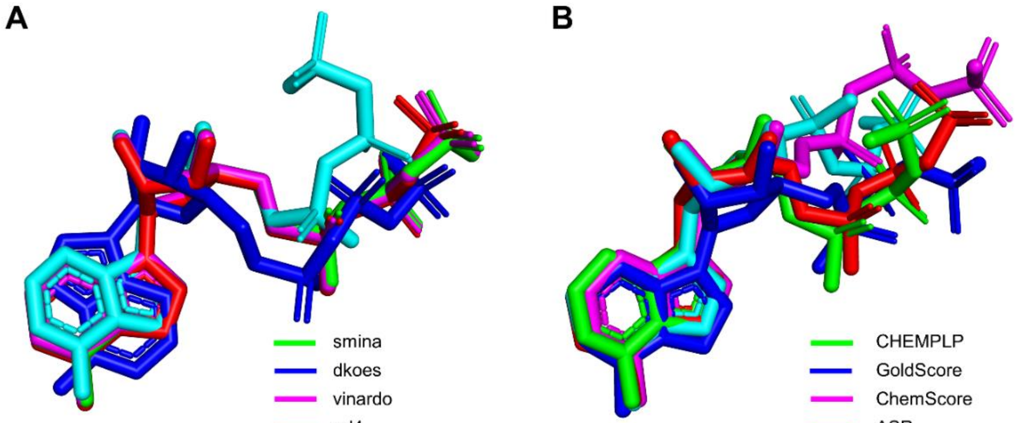


**Figure S4.** Comparison of the experimental ANP conformation (red) and its re-docking pose predicted by **(A)** SMINA docking by smina (green), dkoes (blue), vinardo (magenta), and ad4 (cyan) scoring functions; and **(B)** GOLD docking by CHEMPLP (green), GoldScore (blue), ChemScore (magenta), and ASP (cyan) scoring functions.

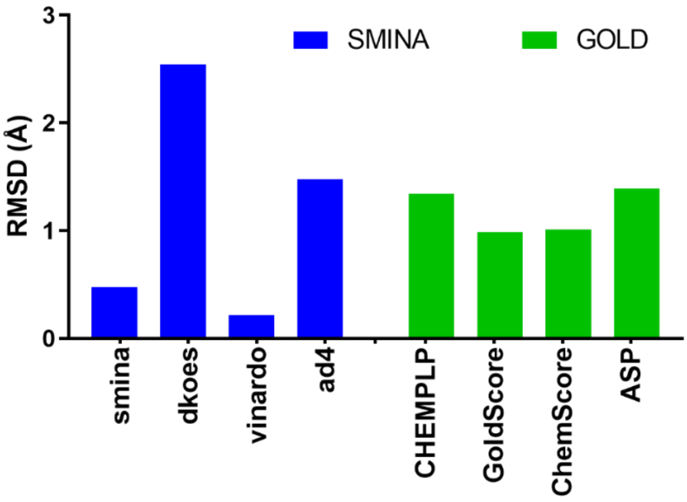


**Figure S5.** The root mean square deviation (RMSD) values between experimental pose of ANP and its docking pose predicted by SMINA with the scoring functions (blue) and GOLD with the scoring functions (green).

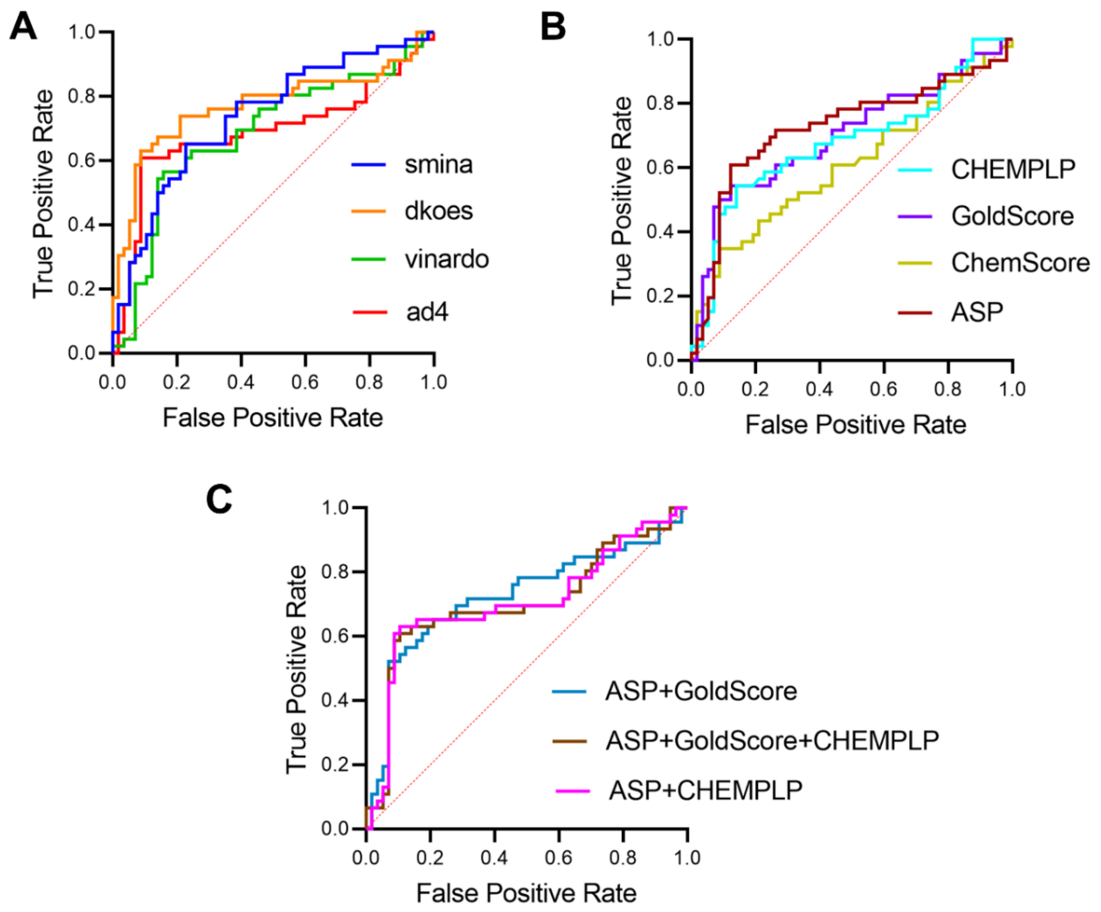


**Figure S6.** The ROC curves of p38γ dataset run by **(A)** SMINA docking with smina (blue), dkoes (orange), vinardo (green), and ad4 (red) scoring functions; **(B)** GOLD docking with CHEMPLP (cyan), GoldScore (violet), ChemScore (olive), and ASP (crimson) scoring functions; and **(C)** GOLD docking with ASP+GoldScore (turquoise), ASP+GoldScore+CHEMPLP (brown), ASP+CHEMPLP (magenta) consensus scoring.

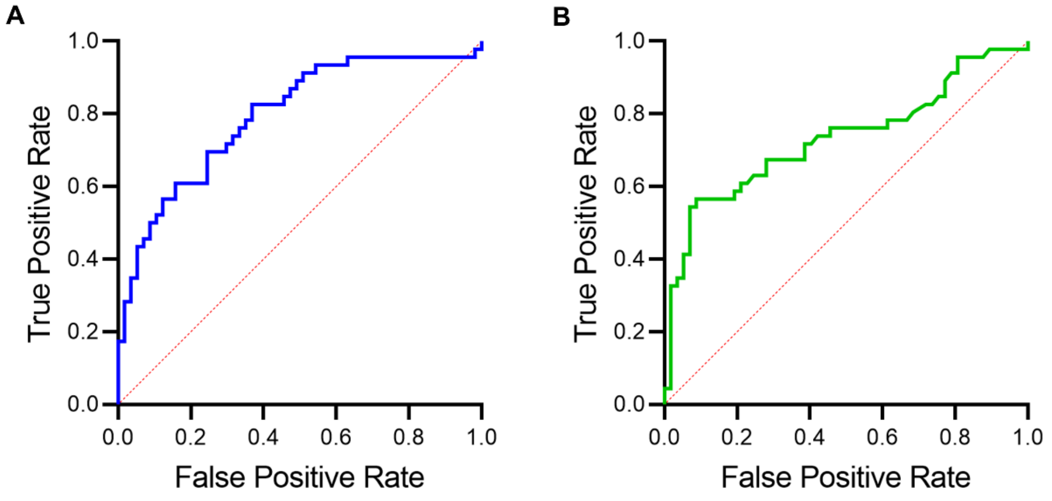


**Figure S7.** Receiver operating characteristic (ROC) curves of the query verification for **(A)** ROCS matching; **(B)** EON comparison.

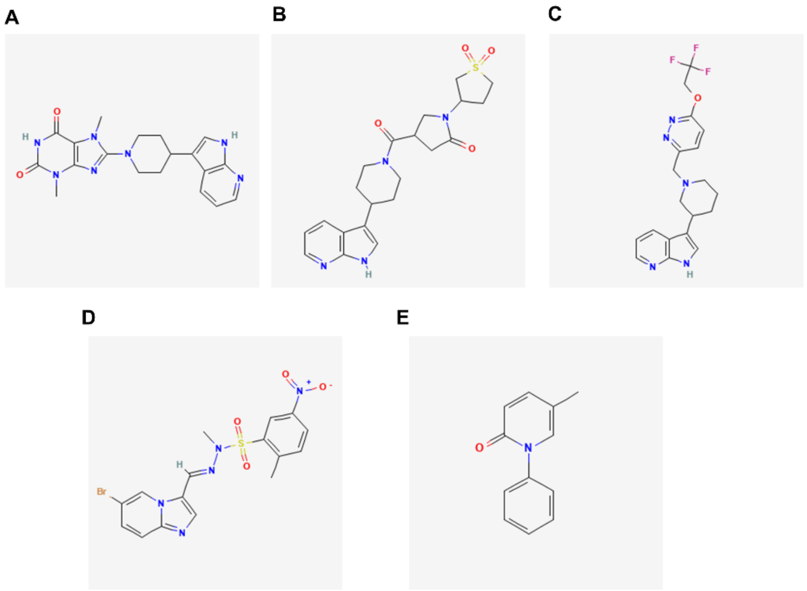


**Figure S8.** 2D chemical structure of **(A)** compound **1; (B)**compound **2; (C)** compound **3; (D)** pirfenidone; and **(E)** PIK75.

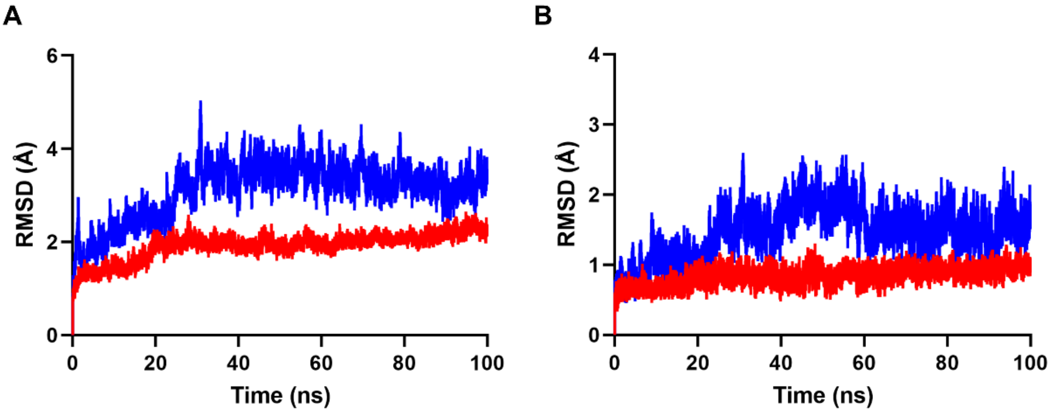


**Figure S9.** Root mean square deviation (RMSD) plots of (**A**) protein backbone; (**B**) binding sites of protein during 100 ns MD simulations. Red stands for compound **2** complex; blue stands for apo protein.

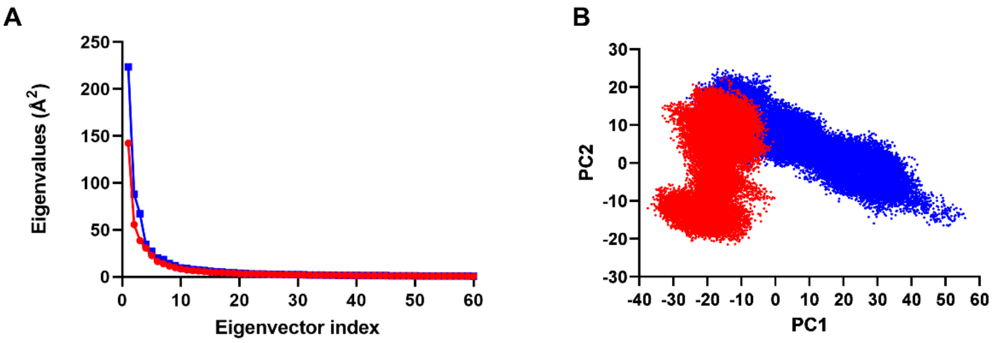


**Figure S10.** (**A**) Eigenvalues of the first 60 eigenvectors derived from PCA of each simulation trajectories of p38γ protein. (**B**) Projection of the motion of combined trajectories along the PC1 and PC2. Red stands for compound **2** complex; blue stands for apo protein.

**Table S1.** Performance summary of QSAR models for predicting $pIC_{50}$.

| Fingerprints | Models | | Training set | 10-fold CV | Test set |
|---|---|---|---|---|---|
| CDK Extended | SVM | $R^2$ | 0.8228 | 0.7491 | 0.7475 |
| | | MAE | 0.2593 | 0.3261 | 0.4016 |
| | | RMSE | 0.3999 | 0.4756 | 0.5260 |
| | RF | $R^2$ | 0.9183 | 0.6277 | 0.7933 |
| | | MAE | 0.1931 | 0.3930 | 0.3788 |
| | | RMSE | 0.2830 | 0.5788 | 0.4767 |
| | RBFNetwork | $R^2$ | 0.6790 | 0.6285 | 0.6637 |
| | | MAE | 0.4098 | 0.4460 | 0.4914 |
| | | RMSE | 0.5347 | 0.5762 | 0.6049 |
| | AdaBoost | $R^2$ | 0.7543 | 0.5361 | 0.6642 |
| | | MAE | 0.3420 | 0.4840 | 0.4793 |
| | | RMSE | 0.4715 | 0.6881 | 0.6434 |
| | MLP | $R^2$ | 0.9172 | 0.3418 | 0.7044 |
| | | MAE | 0.2259 | 0.7035 | 0.5461 |
| | | RMSE | 0.2952 | 1.0353 | 0.6702 |
| | kNN | $R^2$ | 0.9497 | 0.5319 | 0.6727 |
| | | MAE | 0.0904 | 0.4155 | 0.4315 |
| | | RMSE | 0.2116 | 0.6744 | 0.6112 |
| PubChem | SVM | $R^2$ | 0.7222 | 0.6618 | 0.8084 |
| | | MAE | 0.3991 | 0.4316 | 0.4350 |
| | | RMSE | 0.5102 | 0.5565 | 0.4972 |
| | RF | $R^2$ | 0.8325 | 0.6072 | 0.8651 |
| | | MAE | 0.2286 | 0.4174 | 0.2611 |
| | | RMSE | 0.3862 | 0.5992 | 0.3901 |
| | RBFNetwork | $R^2$ | 0.5507 | 0.4525 | 0.7218 |
| | | MAE | 0.4750 | 0.5521 | 0.4718 |
| | | RMSE | 0.6326 | 0.6998 | 0.5804 |
| | AdaBoost | $R^2$ | 0.7295 | 0.6550 | 0.7944 |
| | | MAE | 0.3642 | 0.4104 | 0.4097 |
| | | RMSE | 0.4910 | 0.5569 | 0.4948 |
| | MLP | $R^2$ | 0.7820 | 0.5589 | 0.5316 |
| | | MAE | 0.3360 | 0.5453 | 0.6079 |
| | | RMSE | 0.4457 | 0.6882 | 0.8911 |
| | kNN | $R^2$ | 0.8825 | 0.6343 | 0.7350 |
| | | MAE | 0.2286 | 0.4037 | 0.3835 |
| | | RMSE | 0.3862 | 0.5735 | 0.5631 |
| Klekota-Roth | SVM | $R^2$ | 0.7068 | 0.6487 | 0.7039 |
| | | MAE | 0.4081 | 0.4483 | 0.4986 |
| | | RMSE | 0.5301 | 0.5762 | 0.5943 |
| | RF | $R^2$ | 0.8512 | 0.6078 | 0.7422 |
| | | MAE | 0.2349 | 0.4043 | 0.4082 |
| | | RMSE | 0.3639 | 0.6011 | 0.5280 |
| | RBFNetwork | $R^2$ | 0.4990 | 0.4858 | 0.6696 |
| | | MAE | 0.5466 | 0.5530 | 0.5463 |
| | | RMSE | 0.6682 | 0.6780 | 0.6303 |
| | AdaBoost | $R^2$ | 0.7210 | 0.5477 | 0.6558 |
| | | MAE | 0.4020 | 0.4821 | 0.5187 |
| | | RMSE | 0.4995 | 0.6438 | 0.6117 |
| | MLP | $R^2$ | 0.8425 | 0.5061 | 0.7271 |
| | | MAE | 0.2613 | 0.5316 | 0.4116 |
| | | RMSE | 0.3819 | 0.7475 | 0.5437 |
| | kNN | $R^2$ | 0.8512 | 0.6482 | 0.7422 |
| | | MAE | 0.2349 | 0.3797 | 0.4082 |
| | | RMSE | 0.3639 | 0.5643 | 0.5280 |

$R^2$: coefficient of determination ($R^2$ for training set, $Q^2$ for cross-validation and test set);
MAE: mean absolute error; and RMSE: root mean squared error.

**Table S2.** Checklist of hit compounds and their predicted information.

| idnumber | Predicted $pIC_{50}$ | Smina scoring | Gold scoring | ROCS Overlap | EON_ET combo | Solubility ForecastIndex | 4_400 | 3_75 | Fsp3 | Functionnal Group | FAF Result | Aggregates Category | PAINS Filter | promiscuous pScore |
|---|---|---|---|---|---|---|---|---|---|---|---|---|---|---|
| **Z1587203987** | **6.2438** | **-10.861** | **0.547** | **1063.880** | **0.735** | **Good** | **good** | **good** | **0.42** | **No** | **Accepted** | **0** | **0** | **21** |
| **Z2951708795** | **6.3213** | **-9.402** | **1.709** | **943.267** | **0.587** | **Good** | **good** | **good** | **0.42** | **Yes** | **Accepted** | **0** | **0** | **272** |
| **Z806431270** | **6.3883** | **-9.686** | **0.468** | **930.631** | **0.377** | **Good** | **good** | **good** | **0.57** | **No** | **Accepted** | **0** | **0** | **223** |
| Z1033427004 | 6.2252 | -9.561 | 3.694 | 874.239 | 0.370 | Reduced | bad | warning | 0.15 | Yes | Intermediate | 0 | 0 | 370 |
| Z1084264954 | 6.0282 | -9.575 | 0.732 | 938.455 | 0.351 | Good | good | good | 0.41 | No | Accepted | 0 | 0 | 258 |
| Z1101148148 | 6.0698 | -9.791 | 2.296 | 809.049 | 0.450 | Reduced | bad | warning | 0.31 | Yes | Intermediate | 0 | 0 | 328 |
| Z1183550855 | 6.2462 | -10.117 | 0.674 | 918.778 | 0.331 | Good | good | good | 0.36 | No | Accepted | 0 | 0 | 328 |
| Z1184505037 | 6.0255 | -9.328 | 0.283 | 823.848 | 0.335 | Reduced | good | bad | 0.18 | Yes | Intermediate | 0 | 0 | 328 |
| Z1225312254 | 6.2252 | -9.010 | 0.356 | 892.712 | 0.770 | Reduced | good | warning | 0.39 | No | Accepted | 1 | 0 | 370 |
| Z1261379827 | 6.1635 | -9.164 | 0.633 | 860.137 | 0.325 | Reduced | good | good | 0.45 | Yes | Intermediate | 0 | 0 | 328 |
| Z1262980692 | 6.0941 | -9.150 | 0.849 | 997.905 | 0.644 | Reduced | good | bad | 0.39 | No | Accepted | 0 | 0 | 98 |
| Z1269870281 | 6.0532 | -9.630 | 1.091 | 891.903 | 0.701 | Good | good | bad | 0.38 | No | Accepted | 0 | 0 | 370 |
| Z1309479708 | 6.0001 | -9.180 | 2.419 | 884.599 | 0.675 | Reduced | good | warning | 0.39 | Yes | Rejected | 1 | 0 | 172 |
| Z1341880655 | 6.4037 | -9.750 | 2.264 | 834.350 | 0.343 | Good | good | good | 0.26 | No | Accepted | 0 | 0 | 0 |
| Z1401106939 | 6.4677 | -9.864 | 0.675 | 858.493 | 0.511 | Good | good | good | 0.39 | No | Accepted | 0 | 0 | 98 |
| Z1444992575 | 6.2112 | -10.021 | 1.328 | 810.817 | 0.616 | Reduced | bad | bad | 0.19 | Yes | Intermediate | 0 | 0 | 370 |
| Z1482020288 | 6.4397 | -9.872 | 2.842 | 838.686 | 0.389 | Reduced | good | warning | 0.2 | Yes | Intermediate | 0 | 0 | 333 |
| Z1563314092 | 6.0128 | -9.400 | 1.188 | 817.842 | 0.732 | Good | good | good | 0.45 | No | Accepted | 0 | 0 | 328 |
| Z1657305548 | 6.3565 | -9.499 | 0.162 | 805.224 | 0.684 | Good | good | warning | 0.57 | No | Accepted | 1 | 0 | 328 |
| Z1699662493 | 6.1886 | -9.932 | 2.934 | 856.442 | 0.369 | Good | good | good | 0.42 | No | Accepted | 0 | 0 | 26 |
| Z1866911854 | 6.0080 | -9.567 | 0.956 | 828.068 | 0.359 | Good | good | good | 0.42 | Yes | Intermediate | 0 | 0 | 219 |
| Z1869745863 | 6.0103 | -9.606 | 0.445 | 852.566 | 0.780 | Good | good | warning | 0.45 | No | Accepted | 0 | 0 | 328 |
| Z1886893508 | 6.4136 | -10.826 | 1.469 | 914.080 | 0.647 | Good | good | good | 0.3 | No | Accepted | 1 | 0 | 194 |
| Z1908694822 | 6.3406 | -9.451 | 1.302 | 846.252 | 0.353 | Reduced | good | good | 0.45 | No | Accepted | 0 | 0 | 328 |
| Z1915974655 | 6.0659 | -9.772 | 1.479 | 848.800 | 0.729 | Reduced | good | bad | 0.4 | No | Accepted | 0 | 0 | 370 |
| Z1919132892 | 6.2992 | -9.369 | 0.605 | 957.565 | 0.340 | Good | good | warning | 0.57 | Yes | Intermediate | 0 | 0 | 292 |
| Z1972676030 | 6.0407 | -9.461 | 2.960 | 982.852 | 0.379 | Good | good | good | 0.5 | No | Accepted | 0 | 0 | 439 |
| Z1981843863 | 6.5277 | -9.638 | 1.369 | 887.057 | 0.551 | Good | good | good | 0.44 | No | Accepted | 0 | 0 | 105 |

| idnumber | Predicted $pIC_{50}$ | Smina scoring | Gold scoring | ROCS Overlap | EON_ET combo | Solubility ForecastIndex | 4_400 | 3_75 | Fsp3 | Functionnal Group | FAF Result | Aggregates Category | PAINS Filter | promiscuous pScore |
|---|---|---|---|---|---|---|---|---|---|---|---|---|---|---|
| Z2004784913 | 6.3737 | -9.162 | 0.318 | 928.296 | 0.404 | Good | good | good | 0.76 | No | Accepted | 0 | 0 | 292 |
| Z2019179059 | 6.0659 | -9.367 | 1.681 | 874.998 | 0.378 | Reduced | good | bad | 0.4 | No | Accepted | 0 | 0 | 370 |
| Z2201387782 | 6.2112 | -10.157 | 0.276 | 876.602 | 0.452 | Reduced | good | warning | 0.33 | Yes | Intermediate | 0 | 0 | 370 |
| Z2272277236 | 6.0095 | -9.913 | 3.160 | 899.615 | 0.736 | Reduced | good | good | 0.29 | No | Accepted | 0 | 0 | 439 |
| Z2433619273 | 6.1450 | -9.528 | 1.791 | 962.904 | 0.679 | Good | good | warning | 0.38 | No | Accepted | 0 | 0 | 370 |
| Z2447455936 | 6.0659 | -9.051 | 1.798 | 825.248 | 0.505 | Good | good | bad | 0.35 | No | Accepted | 0 | 0 | 370 |
| Z2447456066 | 6.0659 | -9.585 | 2.629 | 840.705 | 0.577 | Reduced | good | good | 0.35 | No | Accepted | 0 | 0 | 370 |
| Z245305452 | 6.0659 | -9.332 | 2.311 | 820.891 | 0.859 | Reduced | bad | bad | 0.4 | No | Accepted | 0 | 0 | 370 |
| Z2719987102 | 6.0683 | -9.120 | 0.172 | 858.602 | 0.347 | Good | good | good | 0.29 | Yes | Intermediate | 0 | 0 | 328 |
| Z2783585812 | 6.1624 | -10.305 | 0.633 | 971.589 | 0.515 | Good | good | good | 0.25 | Yes | Intermediate | 0 | 0 | 328 |
| Z2902784717 | 6.0324 | -9.392 | 3.726 | 980.722 | 0.478 | Reduced | good | good | 0.23 | Yes | Intermediate | 0 | 0 | 597 |
| Z2967330298 | 6.3328 | -9.059 | 0.385 | 1016.329 | 0.680 | Reduced | bad | bad | 0.45 | Yes | Intermediate | 0 | 0 | 0 |
| Z2967330308 | 6.1783 | -9.525 | 2.439 | 942.462 | 0.705 | Reduced | bad | bad | 0.17 | Yes | Intermediate | 0 | 0 | 0 |
| Z2967330466 | 6.0021 | -9.751 | 3.517 | 1025.000 | 0.417 | Reduced | bad | bad | 0 | Yes | Intermediate | 1 | 0 | 636 |
| Z2967331253 | 6.0116 | -9.993 | 1.435 | 1011.658 | 0.451 | Reduced | bad | bad | 0.3 | Yes | Intermediate | 0 | 1 | 906 |
| Z2972447558 | 6.2047 | -9.534 | 1.667 | 815.953 | 0.791 | Good | good | good | 0.89 | No | Accepted | 0 | 0 | 1282 |
| Z2982969327 | 6.0383 | -9.724 | 1.173 | 941.361 | 0.467 | Good | good | good | 0.85 | No | Accepted | 0 | 0 | 269 |
| Z3019090247 | 6.0659 | -9.428 | 1.363 | 864.919 | 0.794 | Good | good | bad | 0.35 | No | Accepted | 0 | 0 | 370 |
| Z3347318836 | 6.0953 | -9.452 | 1.507 | 936.514 | 0.672 | Good | good | good | 0.25 | No | Accepted | 0 | 0 | 193 |
| Z3399557902 | 6.1450 | -9.016 | 4.631 | 846.518 | 0.384 | Reduced | good | warning | 0.19 | Yes | Intermediate | 1 | 0 | 370 |
| Z3601812091 | 6.0672 | -9.013 | 1.760 | 830.513 | 0.347 | Good | good | good | 0.43 | No | Accepted | 0 | 0 | 328 |
| Z3887647047 | 6.1701 | -9.052 | 0.360 | 927.325 | 0.670 | Good | good | good | 0.41 | No | Accepted | 0 | 0 | 98 |
| Z4056455597 | 6.0633 | -9.906 | 3.741 | 914.236 | 0.861 | Reduced | bad | warning | 0.13 | Yes | Intermediate | 1 | 0 | 328 |
| Z4188962652 | 6.0995 | -9.358 | 1.385 | 805.913 | 0.618 | Reduced | good | bad | 0.42 | Yes | Intermediate | 0 | 0 | 263 |
| Z4188962729 | 6.0405 | -9.350 | 2.374 | 964.286 | 0.704 | Reduced | bad | warning | 0.25 | Yes | Rejected | 0 | 0 | 462 |
| Z4235706129 | 6.1703 | -10.143 | 0.600 | 884.514 | 0.377 | Reduced | good | good | 0.2 | No | Accepted | 1 | 0 | 387 |
| Z4353783770 | 6.0659 | -9.992 | 4.108 | 914.541 | 0.679 | Reduced | good | warning | 0.24 | Yes | Intermediate | 0 | 0 | 370 |
| Z4425610538 | 6.1371 | -9.366 | 2.381 | 833.012 | 0.354 | Reduced | good | good | 0.21 | Yes | Intermediate | 0 | 0 | 333 |
| Z4515687684 | 6.4679 | -9.284 | 3.189 | 836.695 | 0.850 | Good | good | good | 0.4 | Yes | Intermediate | 0 | 0 | 370 |
| Z4515700141 | 6.0659 | -9.428 | 2.414 | 863.079 | 0.414 | Reduced | good | bad | 0.35 | No | Accepted | 0 | 0 | 370 |

| idnumber | Predicted $pIC_{50}$ | Smina scoring | Gold scoring | ROCS Overlap | EON_ET combo | Solubility ForecastIndex | 4_400 | 3_75 | Fsp3 | Functionnal Group | FAF Result | Aggregates Category | PAINS Filter | promiscuous pScore |
|---|---|---|---|---|---|---|---|---|---|---|---|---|---|---|
| Z4521554169 | 6.1504 | -9.230 | 0.375 | 833.784 | 0.647 | Good | good | good | 0.59 | No | Accepted | 0 | 0 | 263 |
| Z4521554415 | 6.0143 | -9.983 | 0.856 | 856.567 | 0.699 | Good | good | good | 0.57 | No | Accepted | 0 | 0 | 263 |
| Z4534371556 | 6.0674 | -9.551 | 0.821 | 886.933 | 0.477 | Good | good | good | 0.25 | No | Accepted | 0 | 0 | 161 |
| Z4549063742 | 6.0113 | -9.355 | 4.245 | 817.181 | 0.384 | Reduced | good | good | 0.36 | Yes | Intermediate | 0 | 0 | 328 |
| Z4560504400 | 6.3388 | -9.727 | 2.916 | 831.099 | 0.324 | Good | good | good | 0.11 | Yes | Intermediate | 0 | 0 | 328 |
| Z4661325523 | 6.0154 | -10.042 | 0.988 | 885.484 | 0.384 | Reduced | good | warning | 0.23 | No | Accepted | 0 | 0 | 370 |
| Z4873984150 | 6.1267 | -9.527 | 4.162 | 885.826 | 0.379 | Good | good | warning | 0.52 | No | Accepted | 0 | 0 | 328 |
| Z4898005276 | 6.0311 | -9.827 | 5.898 | 956.884 | 0.760 | Good | good | good | 0.26 | Yes | Intermediate | 1 | 0 | 275 |
| Z4898006235 | 6.2274 | -9.888 | 4.745 | 1025.575 | 0.560 | Reduced | good | warning | 0.54 | No | Intermediate | 0 | 0 | 292 |
| Z49541384 | 6.0142 | -9.098 | 1.979 | 823.621 | 0.440 | Reduced | bad | warning | 0.25 | Yes | Intermediate | 1 | 1 | 597 |
| Z615608400 | 6.2290 | -10.180 | 0.949 | 810.137 | 0.358 | Good | good | good | 0.14 | No | Accepted | 0 | 0 | 535 |
| Z748555870 | 6.0945 | -9.709 | 0.888 | 858.268 | 0.326 | Reduced | good | good | 0.3 | Yes | Intermediate | 1 | 0 | 292 |

The retained molecule form negative design in red. And final 3 hit molecules in bold font.

**Table S3.** 16 hit compounds list.

| ID | mol | Formula | CLogP | MW |
|---|---|---|---|---|
| Z806431270 | 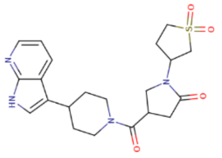 | $C_{21}H_{26}N_4O_4S$ | 0.175 | 430.5 |
| Z1587203987 | 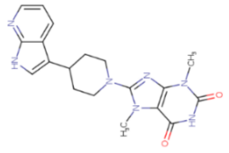 | $C_{19}H_{21}N_7O_2$ | 1.147 | 379.4 |
| Z2951708795 | 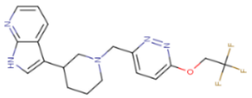 | $C_{19}H_{20}F_3N_5O$ | 2.513 | 391.4 |
| Z3347318836 | 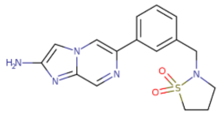 | $C_{16}H_{17}N_5O_2S$ | 1.115 | 343.4 |
| Z1084264954 | 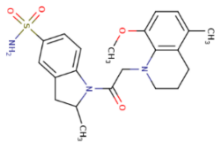 | $C_{22}H_{27}N_3O_4S$ | 3.414 | 429.5 |

| | | | | |
|---|---|---|---|---|
| Z1981843863 | 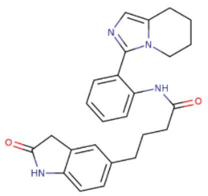 | $C_{25}H_{26}N_4O_2$ | 2.129 | 414.5 |
| Z4521554169 | 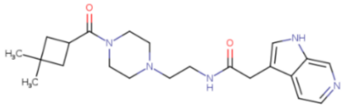 | $C_{22}H_{31}N_5O_2$ | 1.731 | 397.5 |
| Z1401106939 | 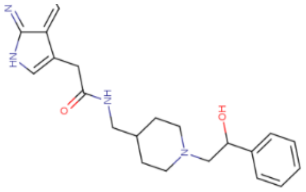 | $C_{23}H_{28}N_4O_2$ | 1.404 | 392.5 |
| Z2982969327 | 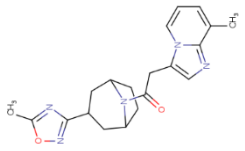 | $CC_1=NC(=NO_1)C_1CC_2CCC(C_1)N_2C(=O)CC_1=CN=C_2N_1C=CC=C_2C$ | 1.897 | 365.4 |
| Z1262980692 | 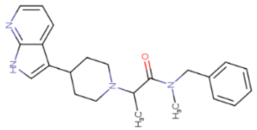 | $C_{23}H_{28}N_4O$ | 3.535 | 376.5 |
| Z3887647047 | 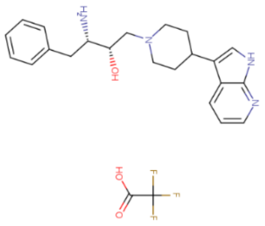 | $C_{24}H_{29}F_3N_4O_3$ | 2.373 | 478.5 |

| | | | | |
|---|---|---|---|---|
| Z2004784913 | 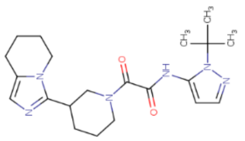 | $C_{21}H_{30}N_6O_2$ | 1.352 | 398.5 |
| Z1699662493 | 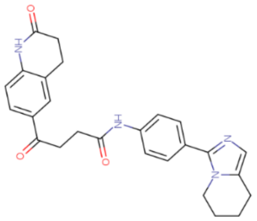 | $C_{26}H_{26}N_4O_3$ | 3.305 | 442.5 |
| Z1341880655 | 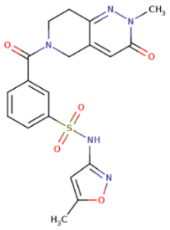 | $C_{19}H_{19}N_5O_5S$ | -0.289 | 429.4 |
| Z4534371556 | 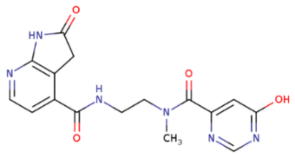 | $C_{16}H_{16}N_6O_4$ | -0.764 | 356.3 |
| Z4898006235 | 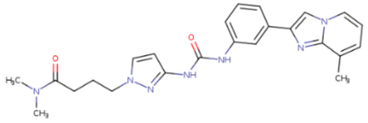 | $C_{24}H_{27}N_7O_2$ | 3.253 | 445.5 |

**Table S4.** The values of RMSD and Rg, and number of hydrogen bonds of systems during simulation.

| Component | compound 1 | compound 2 | compound 3 | pirfenidone | PIK-75 |
|---|---|---|---|---|---|
| RMSD of protein backbone (Å) | | | | | |
| Average | 2.5200 | 1.9139 | 3.0612 | 2.0497 | 2.1366 |
| Max | 3.7370 | 2.6525 | 4.3087 | 3.0809 | 2.7841 |
| Min | 0.5025 | 0.5382 | 0.5592 | 0.5853 | 0.5522 |
| RMSD of ligand (Å) | | | | | |
| Average | 2.2913 | 1.1274 | 1.1040 | 0.7028 | 1.6830 |
| Max | 2.8507 | 1.7808 | 2.026 | 1.4717 | 2.5418 |
| Min | 0.2833 | 0.3196 | 0.2521 | 0.1514 | 0.3736 |
| RMSD of binding site (Å) | | | | | |
| Average | 1.2624 | 0.8391 | 1.9604 | 1.1574 | 1.1522 |
| Max | 2.2780 | 1.3064 | 2.6693 | 1.8917 | 1.6976 |
| Min | 0.3096 | 0.3161 | 0.4387 | 0.3892 | 0.4085 |
| Rg of protein backbone (Å) | | | | | |
| Average | 21.8465 | 21.6577 | 22.1780 | 21.5935 | 21.7364 |
| Max | 22.6247 | 22.1568 | 22.8107 | 22.1296 | 22.4901 |
| Min | 21.4186 | 21.1788 | 21.2531 | 21.1188 | 21.3013 |
| Difference | 1.2061 | 0.978 | 1.5576 | 1.0108 | 1.1888 |
| Rg of ligand (Å) | | | | | |
| Average | 4.7222 | 5.1217 | 3.9898 | 2.6715 | 3.6138 |
| Max | 4.9542 | 5.4913 | 4.5887 | 2.7585 | 4.1418 |
| Min | 8.7312 | 4.4561 | 3.5018 | 2.5846 | 3.1218 |
| Difference | 0.7338 | 1.0352 | 1.0869 | 0.1739 | 1.0200 |
| Rg of binding site (Å) | | | | | |
| Average | 9.2212 | 8.9084 | 9.3855 | 9.1045 | 9.1133 |
| Max | 9.7025 | 9.3337 | 9.8790 | 9.8441 | 9.6939 |
| Min | 8.7312 | 8.6564 | 8.9023 | 8.7023 | 8.7520 |
| Difference | 0.9713 | 0.6773 | 0.9767 | 1.1418 | 0.9419 |
| Number of FON hydrogen bonds between ligand and protein | | | | | |
| Max | 4 | 5 | 3 | 2 | 4 |
| occupancy>20% | 1 | 3 | 2 | 2 | 2 |
